# Nek7 conformational flexibility and inhibitor binding probed through protein engineering of the R-spine

**DOI:** 10.1042/BCJ20200128

**Published:** 2020-04-29

**Authors:** Matthew J. Byrne, Nazia Nasir, Christine Basmadjian, Chitra Bhatia, Rory F. Cunnison, Katherine H. Carr, Corine Mas-Droux, Sharon Yeoh, Céline Cano, Richard Bayliss

**Affiliations:** 1 Astbury Centre for Structural Molecular Biology, School of Molecular and Cellular Biology, Faculty of Biological Sciences, University of Leeds, Leeds, U.K.; 2 Newcastle University Centre for Cancer, School of Natural and Environmental Sciences, Newcastle University, Newcastle Upon Tyne, U.K.; 3 Department of Molecular and Cell Biology, University of Leicester, Leicester, U.K.; 4 Section of Structural Biology, The Institute of Cancer Research, London, U.K.

**Keywords:** kinases, protein engineering, small molecules

## Abstract

Nek7 is a serine/threonine-protein kinase required for proper spindle formation and cytokinesis. Elevated Nek7 levels have been observed in several cancers, and inhibition of Nek7 might provide a route to the development of cancer therapeutics. To date, no selective and potent Nek7 inhibitors have been identified. Nek7 crystal structures exhibit an improperly formed regulatory-spine (R-spine), characteristic of an inactive kinase. We reasoned that the preference of Nek7 to crystallise in this inactive conformation might hinder attempts to capture Nek7 in complex with Type I inhibitors. Here, we have introduced aromatic residues into the R-spine of Nek7 with the aim to stabilise the active conformation of the kinase through R-spine stacking. The strong R-spine mutant Nek7^SRS^ retained catalytic activity and was crystallised in complex with compound **51**, an ATP-competitive inhibitor of Nek2 and Nek7. Subsequently, we obtained the same crystal form for wild-type Nek7^WT^ in apo form and bound to compound **51**. The R-spines of the three well-ordered Nek7^WT^ molecules exhibit variable conformations while the R-spines of the Nek7^SRS^ molecules all have the same, partially stacked configuration. Compound **51** bound to Nek2 and Nek7 in similar modes, but differences in the precise orientation of a substituent highlights features that could be exploited in designing inhibitors that are selective for particular Nek family members. Although the SRS mutations are not required to obtain a Nek7–inhibitor structure, we conclude that it is a useful strategy for restraining the conformation of a kinase in order to promote crystallogenesis.

## Introduction

The NIMA-related kinases (Neks) are a family of Ser/Thr protein kinases, first discovered in *Aspergillus nidulans* that are conserved in eukaryotes [1]. Nek7 is 1 of 11 Neks found in humans, and contributes to the assembly of a robust mitotic spindle apparatus [2,3]. The mitotic substrates of Nek7 include the regulator of G-protein signalling RGS2 [4] and the microtubule-binding protein EML4 [5]. Recent work has expanded our view of Nek7's role to a wider range of cellular pathways. For example, Nek7 mediates the assembly of the NLRP3 inflammasome, a protein complex that activates inflammatory caspases in response to microbes [6–9]. Nek7 also contributes to the formation of microtubule-dependent, extended cytoplasmic protrusions associated with the motility of non-small cell lung cancer cells that express the EML4-ALK fusion protein [10]. Thus, Nek7 activity contributes to several disease-related pathways and is a potential target for therapeutic intervention.

Human Nek7 is a 34.5 kDa protein, comprising a singular canonical kinase domain made up of a small, 5 β-stranded, N-terminal lobe linked to a larger, mostly α-helical C-terminal lobe. The intervening cleft between the two lobes forms the ATP-binding pocket and active site in which phosphoryl transfer takes place. The crystal structure of unphosphorylated, inactive Nek7 revealed an autoinhibited conformation characterised by the orientation of the side chain of Tyr97 pointing down into the active site of the kinase [11]. The binding of Nek9 to Nek7 induces back-to-back dimerisation, which releases autoinhibition and activates the kinase. Autoinhibition of Nek6 and Nek7 can also be overcome by mutation of Tyr97 to Phe, resulting in loss of cell-cycle regulation and dependence on Nek9 [12]. Analysis of the structures of protein kinases revealed two ‘hydrophobic spines’, regulatory (R-spine) and catalytic (C-spine), which traverse the N and C-lobes [13]. The autoinhibitory Tyr97 of Nek7 is one of four R-spine residues, and the mechanism of Nek7 regulation is thus a variation on a common theme among kinases [14]. There is a closely related Nek7 paralogue called Nek6, which shares ∼86% sequence identity, and differs by only 1 of 28 predicted active site residues. It would, therefore, appear to be challenging to develop an ATP-competitive inhibitor that discriminates between the two kinases. Despite this high degree of sequence (and presumably structural) homology, Nek6 and Nek7 have different protein substrate preferences and are not functionally redundant [15]. However, the key features of the regulation of Nek6 and Nek7 are conserved, such as the autoinhibitory Tyr side chain and the binding site for Nek9. Although the activation mechanisms of Nek6 and Nek7 have been characterised, there is no structure of these kinases, nor any Nek family member, in an active conformation.

Studies on the cellular functions of protein kinases are underpinned by the availability of kinase inhibitors [16]. Unfortunately, the Nek family of kinases suffers from a shortage of such tools and, with the exception of Nek2, there are no published attempts to develop selective and potent inhibitors of Neks [17–21]. This situation is likely to change over the next few years because the important biological roles of this family have been identified. However, screening of known kinase inhibitors against panels of human kinases typically finds Nek7 as one of the lowest frequency hits, and therefore there are relatively few promising starting points for the development of Nek7 inhibitors [22]. Furthermore, although the crystal structure of Nek7 was determined in 2009 there are still no crystal structures of Nek7 bound to an inhibitor, which limits the scope for structure-guided design of inhibitors. Nek7 was first crystallised in the absence of ligand, resulting in crystals of space group *I222* that diffracted to 2.1 Å resolution. These crystals were soaked with ADP, which had only a modest impact on resolution (2.3 Å). This appeared to be a promising crystal system upon which to base a structure-guided approach to the development of Nek7 inhibitors. However, the reproducibility of these crystals was extremely poor — *de novo* nucleation of crystals was rare and attempts to generate crystals via seeding methods also failed. In the presence of a short peptide from Nek9, Nek7 forms crystals in a *P3_1_21* space group that diffract to 2.78 Å [12]. Although these crystals are highly reproducible, attempts to co-crystallise with Nek7 under these conditions have also failed in our hands.

We, therefore, set out to identify new starting points for Nek7 inhibitors and to obtain a crystal structure of the Nek7–inhibitor complex. We screened a library of ATP-competitive, Type I Nek2 inhibitors in co-crystallisation trials with Nek7, which yielded the crystal structure of Nek7 bound to a Type I inhibitor. We also determined the structure of Nek2 bound to the same inhibitor, to enable a direct comparison of binding modes. These structures provide insights into the differences between Nek family members that might aid in the development of chemical inhibitors.

## Materials and methods

### Chemical synthesis

Compound **51** was synthesised as described previously [21].

### Protein production and crystallisation

Nek7 strong R-spine mutant (Nek7^SRS^: L86H Y97F L180F) was generated using the Quikchange method from a wild-type Nek7 pET-30 (Novagen) construct encoding a C-terminal non-cleavable His_6_-tag. The resultant plasmid was sequenced to confirm the presence of desired mutations.

Nek7^WT^ and Nek7^SRS^ for crystallisation experiments were co-expressed with lambda phosphatase as previously described for wild-type Nek7 to generate homogeneous, unphosphorylated samples [12,23]. Nek7^WT^ and Nek7^SRS^ used for kinase assays were produced in *Escherichia coli* in the absence of lambda phosphatase to enable autoactivation through autophosphorylation [24]. *E. coli* cell pellets containing recombinantly expressed Nek7^SRS^ or Nek7^WT^ were lysed by sonication in lysis buffer containing 20 mM imidazole 50 mM HEPES (pH 7.5), 300 mM NaCl, 1 mM MgCl_2_ (Nek7^SRS^) or 50 mM HEPES; pH 7.5, 300 mM NaCl, 5% glycerol, 1 mM MgCl_2_, 0.2 mM MnCl_2_ and a protease inhibitor tablet (Nek7^WT^). The clarified lysate was applied to a 5 ml HisTrap column, pre-charged with nickel, and eluted by FPLC across a gradient of 20–250 mM imidazole. The eluate corresponding to recombinant Nek7 was concentrated and applied to a Superdex 200 16/600 size exclusion column for further purification and buffer exchange, equilibrated in 50 mM HEPES; pH 7.5, 300 mM NaCl, 5% glycerol and 5 mM DTT. The fraction corresponding to the monomer size was pooled, concentrated and analysed using SDS–PAGE.

Initial crystallisation screens were carried out with commercially available screening kits using the sitting drop vapour diffusion method. Protein crystals used for structure solution were obtained using the sitting drop vapour diffusion method. A solution containing 250 µM Nek7^SRS^ and 2.5 mM compound **51** was mixed in a 1 : 1 ratio with well buffer comprising 0.02 M sodium/potassium phosphate, 0.1 M Bis-Tris propane pH 6.5, 20% (w/v) PEG 3350. Crystals were harvested in appropriately sized Litholoops (Molecular Dimensions), washed with a cryoprotectant solution containing 0.02 M sodium/potassium phosphate, 0.1 M Bis-Tris propane pH 6.5, 20% (w/v) PEG 3350 and 20% (v/v) ethylene glycol, and flash-cooled with liquid nitrogen. Nek7^WT^ crystals were obtained in a drop containing 420 µM protein with 20% (w/v) PEG 3350, 150 mM di-sodium DL-malate pH 7.0 screen buffer, in a 1 : 1 ratio. The crystals were harvested a week after the first observation using cryo-loops, washed in cryoprotectant containing 25% glycerol in the screen buffer and flash frozen in liquid nitrogen. Nek7^WT^.**51** crystals were obtained by co-crystallisation. Five-hundred micromolar of Nek7^WT^ protein was incubated with 0.5 mM compound **51** for 30 min. The sitting drop vapour diffusion method was used to set up crystallisation in 1 : 1 protein : buffer ratio. The buffer consisted of 20% (w/v) PEG 3350, 150 mM di-sodium DL-malate; pH 7.0, 3% (w/v) 1,6-hexanediol. The crystals were then harvested a week later, washed in cryoprotectant containing 25% glycerol in the screen buffer and flash-cooled in liquid nitrogen.

Recombinant Nek2 kinase domain (aa 1–271) was expressed, purified and crystallised as previously described [23].

### Crystallographic data collection and processing

Diffraction data were collected at Diamond Light Source on beamlines I04 (Nek7^SRS^.**51**), I03 (Nek7^WT^) and I24 (Nek7^WT^.**51**), and at ESRF on beamline ID23-1 (Nek2.**51**). Nek7^SRS^.**51** data were indexed and reduced using iMosflm [25], scaled and merged using Aimless [26], in the CCP4 software package [27]. Nek7^WT^ and Nek7^WT^.51 data were processed using the autoprocess pipelines at Diamond Light Source, Xia2 Dials [28] and StarAnsio [29], respectively. Nek2.**51** data were indexed and reduced using iMosflm, scaled and merged using Scala in the CCP4 software package. Molecular replacement of Nek7^SRS^.**51** was carried in Phaser [30] using the Nek7^WT^.ADP structure (PDB code 2WQN) as the search model. Iterative rounds of model building and refinement were carried out using Coot and Refmac5, respectively [31,32]. The structure of Nek7^SRS^.**51** at a provisional stage of refinement (PDB code 6GT1) was used as a search model for molecular replacement in the Nek7^WT^ and Nek7^WT^.**51** data. The Nek2.**51** structure was determined using Nek2.ADP as a search model for molecular replacement (PDB code 2W5A). Ligand model building and restraint calculation were carried out using eLBOW in the Phenix crystallography software package [33]. Figures containing crystal structures were prepared using PyMOL.

### *In vitro* kinase assays

Kinase assays were carried out with a LabChip EZ Reader II system (PerkinElmer) at room temperature. The substrate used was a fluorescein-labelled peptide [5-FAM-FLAKSFGSPNRAYKK-CONH2] dissolved in 100 mM HEPES (pH 7.5), 0.003% (v/v) Brij-35, 0.004% (v/v) Tween-20, 10 mM MgCl_2_. Nek7^WT^ (250 nM) or Nek7^SRS^ (1 µM) enzyme was mixed in a 25 µl reaction volume, with 1.5 µM substrate peptide and the stated concentration of compound **51** before the addition of ATP was used to initiate the reaction. Measurements of substrate phosphorylation were taken every 2 min for 1 h. For the calculation for ATP *K*_m_, reaction mixes were prepared as described above with a 3-fold dilution series of ATP. Measurements of substrate phosphorylation were taken every 2 min for 1 h. All assays were carried out in duplicate. Data were plotted using Prism7 (http://www.graphpad.com).

## Results

### Engineering a strong R-spine mutant of Nek7

Based on the similarities between the Nek family members, we reasoned that the library of compounds generated through the Nek2 inhibitor programme might provide starting points for inhibition of Nek7. We took the approach of screening for Nek7/inhibitor complexes via crystallisation. Extensive trials of Nek7 alone and the Nek7/Nek9 complex failed to generate suitable crystals. Nek7 adopts an inactive conformation in all published structures, and we reasoned that Nek7 trapped in an active conformation would crystallise in a different form. Nek7 is activated through phosphorylation of the activation loop on Ser195, and we have previously generated active, phosphorylated Nek7 using genetically encoded phospho-Ser [34]. Unfortunately, we were unable to generate diffraction-quality crystals using this protein. We, therefore, turned to protein engineering to generate an alternative crystal form through manipulation of the conformation of Nek7 by altering the composition of the R-spine.

**Figure 1.**
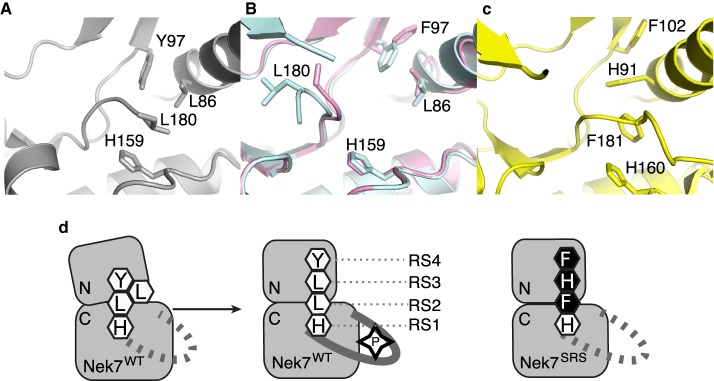
Engineering the R-spine of Nek7 to mimic that of Plk1. (**a**) Collapsed R-spine in the crystal structure of apo-Nek7 (PDB code 2WQM). Side chains of R-spine residues only are shown (RS4-Y97, RS3-L86, RS2-L180, RS1-H159). (**b**) Disrupted R-spine in the crystal structure of Nek7 in complex with Nek9 (PDB code 5DE2). The two chains of Nek7 in the asymmetric unit of the crystal lattice are coloured blue (chain A) and pink (chain B). Note that the protein incorporated the Y97F mutation. (**c**) Assembled R-spine in the crystal structure of Plk1 (PDB code 3D5U). (**d**) Schematic representation of Nek7, showing the proposed stacking of Nek7's R-spine induced by phosphorylation, and the proposed strategy to mimic the strong R-spine of Plk1 through three mutations (Y97F, L86H, L180F).

**Figure 2.**
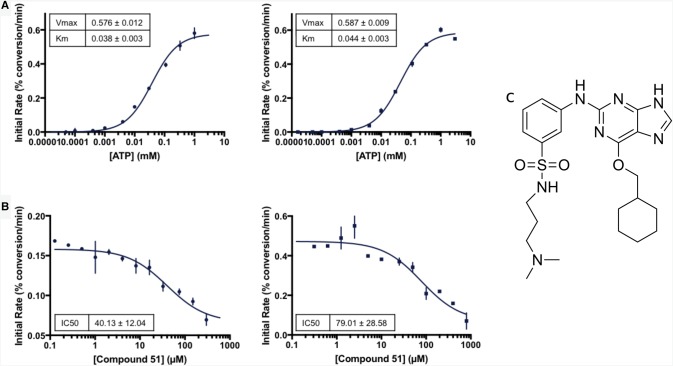
Comparison of Nek7^WT^ and Nek7^SRS^ kinase activity and inhibition. (**a**) Nek7^SRS^ kinase (circles) is catalytically active and retains an ATP *K*_m_ and *V*_max_ comparable to that of Nek7^WT^ (squares). (**b**) Compound **51** inhibits both Nek7^SRS^ (circles) and Nek7^WT^ (squares). (**c**) The chemical structure of compound **51** [21].

The proper stacking of the residues within the R- and C-spines is a hallmark of an active kinase conformation, and is typically triggered by phosphorylation within the activation loop, and binding of the ATP for the R- and C-spines, respectively (Figure 1) [13]. The R-spine comprises two residues in the C-lobe and two residues in the N-lobe, termed RS1–RS4: RS1 is the first residue from the conserved HRD motif (H159 in Nek7); RS2 is the central residue in the DFG motif (L180 in Nek7); RS3 lies in the C-helix (L86 in Nek7); RS4 is at the N-terminal end of the β4 strand (Y97 in Nek7). In all crystal structures of Nek7 currently available in the PDB, the R-spine exhibits a disrupted configuration (PDB codes 2WQM, 2WQN, 5DE2) [11,12], distinct from the stacked configuration found in an active kinase (Figure 1) [34]. In the crystal structure of Nek7^WT^, Tyr97 at position 4 of the R-spine (RS4) formed an autoinhibitory interaction with the activation loop that effectively collapses the R-spine (Figure 1a) [11]. Mutation of Tyr97 to phenylalanine activates Nek7 and Phe97 was released from the autoinhibitory position in one out of two molecules in the crystal structure of a dimer of Nek7^Y97F^ in complex with a peptide derived from Nek9 [12]. Even so, the R-spine of Nek7^Y97F^ has a disrupted configuration (Figure 1b). We supposed that a stacked configuration of the R-spine might be less favoured in Nek7 due to the lack of aromatic stacking in the R-spine which, unusually, has Leu residues at both positions RS3 (L86) and RS2 (L180) (Table 1). In contrast, three out of four members of the Plk family of mitotic Ser/Thr kinases have aromatic residues at all four R-spine positions (Table 1). This may help to stabilise a stacked configuration of the R-spine, consistent with a crystal structure of Plk1 that has a stacked R-spine in the absence of activation loop phosphorylation (Figure 1c) [35]. We were also inspired by the observation that mutation of RS3 from Leu to His is activating in BRAF [36]. Therefore, in an attempt to artificially induce a stacked configuration of the R-spine, we mutated Nek7^Y97F^ to introduce two additional aromatic residues (L86H and L180F) to mimic the R-spine of Plk1 (Table 1 and Figure 1d). We expressed and purified the Nek7 strong R-spine mutant protein (Nek7^SRS^L86H Y97F L180F) and demonstrated that it is catalytically active and retains a similar ATP *K*_m_ and *V*_max_ to that of Nek7^WT^ (Figure 2a).

**Table 1.** Composition of the R-spines from human NEK and PLK family kinases

Kinase	RS4	RS3	RS2	RS1
NEK1	Y	L	F	H
NEK2	Y	L	F	H
NEK3	F	L	F	H
NEK4	Y	L	L	H
NEK5	F	L	F	H
NEK6	Y	L	L	H
NEK7	Y (97)	L (86)	L (180)	H (159)
NEK8	Y	L	F	H
NEK9	Y	L	Y	H
NEK10	Y	I	F	H
NEK11	F	L	F	H
PLK1	F	H	F	H
PLK2	F	H	F	H
PLK3	F	H	F	H
PLK4	L	H	F	H
BRAF	F	L	F	H
NEK7^SRS^	F	H	F	H

**Figure 3.**
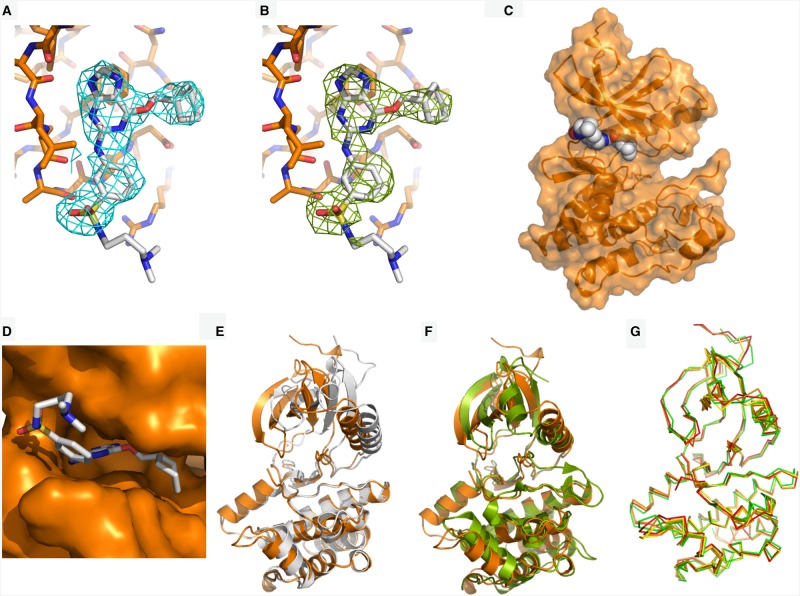
The crystal structure of Nek7^SRS^ bound to compound 51. (**a**) The electron density map (2mFo-DFc) is shown as a cyan wiremesh contoured at 1.0 σ. (**b**) A difference electron density map (mFo-DFc) was generated after removal of compound **51** from the structure and three cycles of PHENIX refinement, including simulated annealing, and is shown as a green wiremesh contoured at 2.5 σ. (**c**) Nek7^SRS^ is coloured orange and shown in cartoon and surface representation. Compound **51** is shown in sphere representation and is coloured grey (carbon), blue (nitrogen), yellow (sulfur) and red (oxygen). (**d**) Compound **51** shown in stick representation, bound in the ATP-binding pocket of Nek7^SRS^, shown in surface representation (orange). (**e**) Superposition of Nek7 bound to compound **51** (orange) with Nek7 bound to ADP (grey). (**f**) Superposition of Nek7^SRS^ bound to compound **51** (orange) and PKA (green, PDB code 1JBP). N- and C-terminal extensions to the kinase domain of PKA are omitted for clarity. (**g**) Superposition of all four chains (A–D) found within the asymmetric unit of Nek7^SRS^ bound to compound **51**. Ligands are omitted for clarity in panels (**e**–**g**).

Dose-response experiments with Nek7^WT^ and Nek7^SRS^ demonstrated that a Nek2 inhibitor, compound **51** [21], successfully inhibited both enzymes (Figure 2b,c). The apparent IC_50_ values calculated in these experiments are 79.01 µM and 40.13 µM for Nek7^WT^ and Nek7^SRS^, respectively. We were unable to obtain crystals of Nek7^SRS^ in the presence of ADP/MgCl_2_, but co-crystallisation screens with Nek7^SRS^ and compound **51** were successful (Figure 3 and Table 2).

**Table 2.** Summary of data collection and refinement statistics for Nek2.51, Nek7^WT^, Nek7^WT^.51 and Nek7^SRS^.51

	Nek2.**51**	Nek7^WT^	Nek7^WT^.**51**	Nek7^SRS^.**51**
PDB code	6SK9	6S76	6S75	6S73
*Data collection*				
Space group	*C 1 2 1*	*P 1 21 1*	*P 1 21 1*	*P 1 21 1*
Cell dimensions				
*a*, *b*, *c* (Å)	100.02 56.89 77.94	50.25 168.06 83.44	50.41 168.97 84.56	49.42, 169.08, 83.73
*α*, *β*, *γ* (°)	90.00 132.42 90.00	90.00 90.00 90.00	90.00 90.03 90.00	90.00, 92.81, 90.00
Wavelength (Å)	0.9795	0.9763	0.9686	0.9795
Resolution (Å)	30.63–2.0 (2.11–2.0)	74.74–3.38 (3.56–3.38)	75.62–3.30 (3.42–3.30)	83.63–3.5 (3.72–3.5)
*R*_merge_	0.087 (0.588)	0.101 (0.347)	0.078 (0.390)	0.176 (0.606)
*I*/σ*I*	8.5 (2.3)	6.5 (2.7)	15.3 (5.4)	4.9 (2.1)
CC_1/2_	0.997 (0.679)	0.986 (0.0.903)	0.999 (0.983)	0.975 (0.645)
Completeness (%)	99.3 (99.9)	99.45 (99.32)	96.44 (79.81)	99.1 (99.30)
Redundancy	3.6 (3.7)	3.3 (3.5)	6.6 (6.9)	3.0 (3.0)
No. of unique reflections measured	21 992	19 383	21 271	17 292
*Refinement*				
Resolution (Å)	30.63–2.0	74.74–3.38	75.62–3.30	83.63–3.50
No. reflections	20 716	19 281	20 549	17 166
*R*_work_/*R*_free_	0.188/0.220	0.262/0.286	0.260/0.317	0.235/0.271
No. atoms				
Protein	1878	7390	7615	7553
Ligand	40	7	102	102
Water	91	—	—	—
*B*-factors (Å^2^)				
Protein	49.4	92.26	49.31	71.3
Ligand	55.7	96.02	57.61	85.63
Water	52.2	—	—	—
r.m.s. deviations				
Bond lengths (Å)	0.008	0.002	0.003	0.002
Bond angles (°)	1.03	0.570	0.552	0.482
*Ramachandran plot*				
Outliers (%)	0.00	0.19	0.29	0.19
Allowed (%)	1.30	9.14	7.63	4.33
Favoured (%)	98.70	90.66	92.08	95.48

The resulting crystal structure shows that compound **51** binds to Nek7^SRS^ in the ATP-binding pocket (PDB code 6S73; Figure 3a–d). Superposition of Nek7^SRS^ bound to compound **51** with Nek7^WT^ (PDB code 2WQN) reveals a ∼10° rotation of the N-lobe relative to the C-lobe (Figure 3e). This moves the β-sheet in the N-lobe by 4–5 Å, relocating the Gly-rich loop to cover the ATP-binding cleft on the C-lobe, effectively restoring its depth to that typical of kinases. Superposition of Nek7^SRS^ bound to compound **51** with PKA in a prototypical active conformation demonstrates that Nek7^SRS^ mutant bound to compound **51** is more similar to the active conformation of a typical kinase than previous structures of Nek7^WT^ or Nek7^Y97F^ (Figure 3f). However, the conformation of Nek7^SRS^ does not exhibit other hallmarks of the kinase active conformation: the DFG motif is not ‘in’, the Lys–Glu salt-bridge (K63/E82) is broken and the activation loop is mostly disordered.

Superposition of each of the four monomers in the asymmetric unit yields RMSDs ranging between 0.5 and 0.7 Å, demonstrating that the monomers have an overall similar conformation (Figure 3g). Despite this, ligands can only be confidently placed in three of four active sites and the quality of side-chain density in chain D is inferior to that of the other three chains. This can be rationalised when inspecting crystal contacts with protomers in adjacent asymmetric units. Chains A–C form many crystal contacts with surrounding protomers whilst chain D has few. Indeed, the density of the N-lobe of chain D was so poor that we modelled only the main chain atoms.

### SRS mutations stabilise the conformation of the R-spine

In the original crystal structure of Nek7^WT^, the side-chain of Y97 points into the active site, and forms a hydrogen bond with the backbone amide of R-spine residue Leu180, acting as an autoinhibitory mechanism. Autoinhibition is released by the mutation Y97F or through the binding of Nek9 [11,12,24]. However, in the structure of the complex between Nek7^Y97F^ and a fragment of Nek9, only one chain of Nek7^Y97F^ shows residue 97 flipped upwards away from the active site. Moreover, the R-spine does not form a stacked configuration, demonstrating that neither the Y97F mutation nor binding of Nek9, nor this mode of back-to-back dimerisation, are sufficient to achieve an active conformation of Nek7.

**Figure 4.**
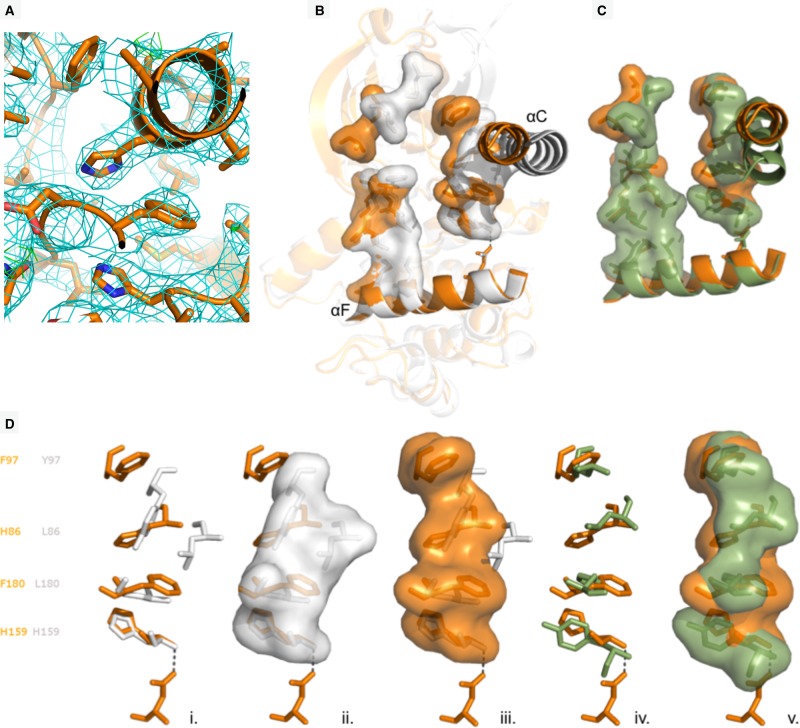
A partially-stacked configuration of the mutated R-spine. (**a**) Structure of Nek7^SRS^.**51** in the R-spine region. The electron density map (2mFo-DFc) is shown as a cyan wiremesh contoured at 1.0 σ. (**b**) Superposition of Nek7^WT^ (grey) with Nek7^SRS^ (orange) shown in cartoon representation with C-helix and F-helix highlighted. C-spine and R-spine residues are shown in stick and surface representation. (**c**) C-spine and R-spine residues of Nek7^SRS^ (orange) superposed with equivalent residues from PKA (green). (**d**) R-spine residues of Nek7^WT^ (grey) with Nek7^SRS^ (orange), from left to right in stick representation, with Nek7^WT^ in stick and surface representation and with Nek7^SRS^ in stick and surface representation. Hydrogen bonds are shown as dashed lines throughout.

The crystal structure of Nek7^SRS^ revealed a configuration of the R-spine in which residues 97 and 86 are shifted laterally compared with previous structures of Nek7^WT^, and are positioned above H159 and F180 (Figure 4a,b). This is broadly similar to the configuration observed in crystal structures of active kinases such as PKA (Figure 4c). However, this is not a correctly assembled R-spine as the side chain of F180 is not in perfect alignment with the other R-spine residues, but is packed against the C-helix (Figure 4a).

**Figure 5.**
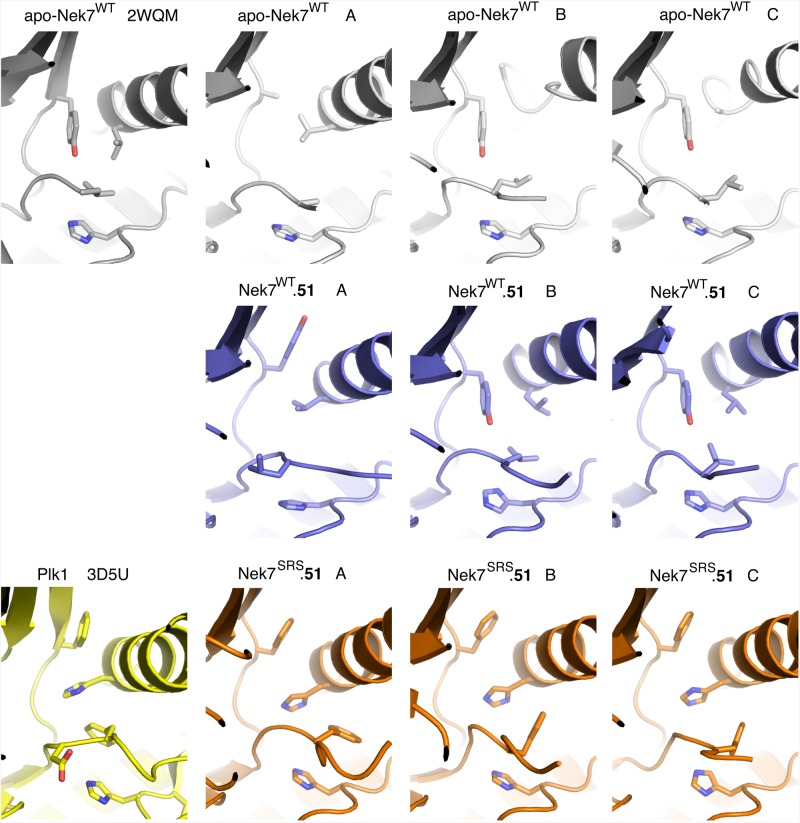
Comparison of the R-spines in crystal structures of Nek7. Views of the R-spines taken from structures of individual Nek7 protein chains. R-spine side chains are shown as sticks.

At this point in the study, we were concerned that the structural changes we observed in Nek7^SRS^ might be a consequence of the presence of the compound or the specific crystal form, not the mutations *per se*. We, therefore, obtained crystals of Nek7^WT^ apo and Nek7^WT^.**51** in the same form as the Nek7^SRS^.**51**. Crystal structures of Nek7^WT^ were determined in apo form (PDB code 6S76) and bound to compound **51** (PDB code 6S75), to limiting resolutions of 3.38 Å and 3.30 Å, respectively, in the same monoclinic crystal form as the Nek7^SRS^.**51** structure (Table 2). All three structures have four chains in the asymmetric unit, and in each case, chain D has a poorly ordered N-lobe. The analysis, therefore, focussed on chains A–C (Figure 5). In chains B and C of the monoclinic apo-Nek7^WT^ and Nek7^WT^.**51** structures, the side chain of Y97 points into the active site, as in the original, orthorhombic Nek7^WT^ structure (PDB code 2WQM). In contrast, the side chain of Y97 adopted the ‘up’ position in chain A of the Nek7^WT^.**51** structure, and was poorly resolved in chain A of the monoclinic Nek7^WT^ structure and was not modelled. These conformational differences might be explained by crystal packing interactions: chains B and C of the monoclinic structures form a symmetric, back-to-back, head-to-tail dimer that covers the surface proximal to Y97, whereas this region of chain A is exposed to solvent.

A comparison of the apo-Nek7^WT^ and Nek7^WT^.**51** structures indicates that the presence of compound **51** does not alter the conformation of the R-spine. Although there might be a stabilising effect of compound **51** on chain A, as the side-chain of Y97 is clear only in the presence of the compound, this might also simply reflect crystal-to-crystal variability. While the monoclinic structures of Nek7^WT^ have two distinct conformations of Y97, all three chains that were fully modelled in the monoclinic structure of Nek7^SRS^**.51** have the same conformation of Y97. This happens despite the different crystal packing interfaces experienced by chain A versus B and C. Therefore, we conclude the partially stacked configuration of the R-spine in the Nek7^SRS^**.51** structure is due to the presence of the mutations, not the compound or crystal packing. The position of L180 in Nek7^WT^ is also more variable than that of F180 in Nek7^SRS^, but the mutant still fails to achieve a fully stacked R-spine as indicated by the position and variability in the side chain of F180 (Figure 5). Nevertheless, the SRS mutation does achieve a more stable and partly stacked R-spine.

### Interaction of compound **51** with Nek7 and Nek2

**Figure 6.**
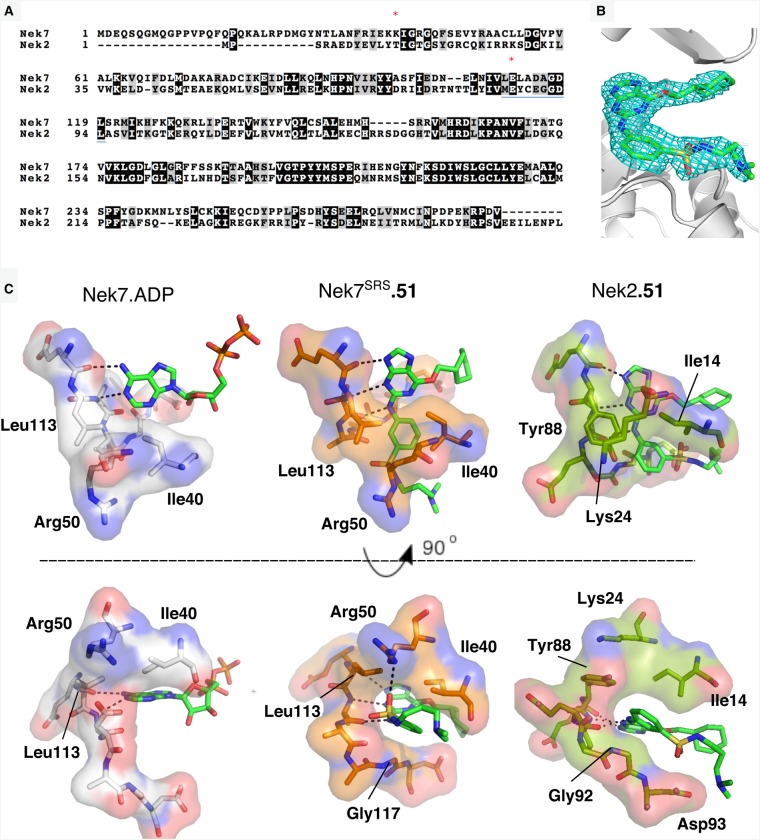
Compound 51 binds to Nek7 and Nek2 with similar binding modes. (**a**) Alignment of human Nek7 and Nek2 sequences. R-spine residues RS3 (Leu88) and RS2 (Leu180) are highlighted with red asterisks and the hinge region is underlined in blue. (**b**) Crystal structure of compound **51** bound to Nek2. The electron density map (2mFo-DFc) is shown as a cyan wiremesh contoured at 1.0 σ. (**c**) Left: Hinge region of Nek7 bound to ADP (grey) shown in stick and surface representation. Middle: Nek7^SRS^ (orange) bound to compound **51** shown in stick and surface representation. Right: Hinge region of Nek2 bound to compound **51** (dark green) shown in stick and surface representation. Hydrogen bonds are shown as dashed black lines. Ligands are coloured as follows: carbon (bright green), nitrogen (blue), oxygen (red), sulfur (yellow) and phosphorus (orange).

Compound **51** inhibits Nek2 with an IC_50_ of 1.42 µM, ∼50-fold more potently than Nek7^SRS^ [21]. To gain insights into this difference, we aligned the protein sequences (Figure 6a) and determined the crystal structure of Nek2 catalytic domain bound to compound **51** to a resolution of 2.0 Å (PDB code 6SK9; Figure 6b). The overall binding mode of compound **51** was similar in both structures: the purine scaffold lines up along the kinase hinge region; the substituted phenyl group sits on a Gly residue in the post-hinge region (G92 in Nek2, G117 in Nek7^SRS^), and the sulphonamide substituent points towards solvent; the cyclohexyl group fits between an Asp residue in the post-hinge region (D93 in Nek2, D118 in Nek7^SRS^) and the Gly-rich loop. As with previous structures of Nek2 bound to purine-based inhibitors, compound **51** forms three H-bonds with the hinge region of Nek2. This is also the case for compound **51** and the hinge region of Nek7^SRS^, via the main chain O of Glu112, the main chain N-H and O of Ala114.

The orientation of the substituted phenyl group of compound **51** is clearly different between the two structures (Figure 6c): when bound to Nek2, the phenyl ring lies flat against Gly92, and the sulphonamide-amine substituent points towards Asp93; when bound to Nek7^SRS^ or Nek7^WT^ the phenyl ring is rotated so that the sulphonamide-amine points towards the N-lobe where it interacts with the side chain of Arg50. The orientation of the sulphonamide-amine substituent of compound **51** bound to Nek7^SRS^ takes advantage of an opening above the hinge region of Nek7 that is not present in Nek2. The equivalent residue to Leu113 of Nek7 in Nek2 is Tyr88, which partly fills the space occupied by the phenyl-sulfonamide of compound **51** in the Nek7-bound pose. Of the human Neks, only Nek6, Nek7 and Nek10 have a Leu at this position, the other 8 Neks have a Tyr or Phe. Thus, the pose of compound **51** when bound to Nek7 reveals a feature that could be explored in the design of inhibitors specific to a subset of Nek7 isoforms.

### Nek2.**51** structure has a weak C-helix

**Figure 7.**
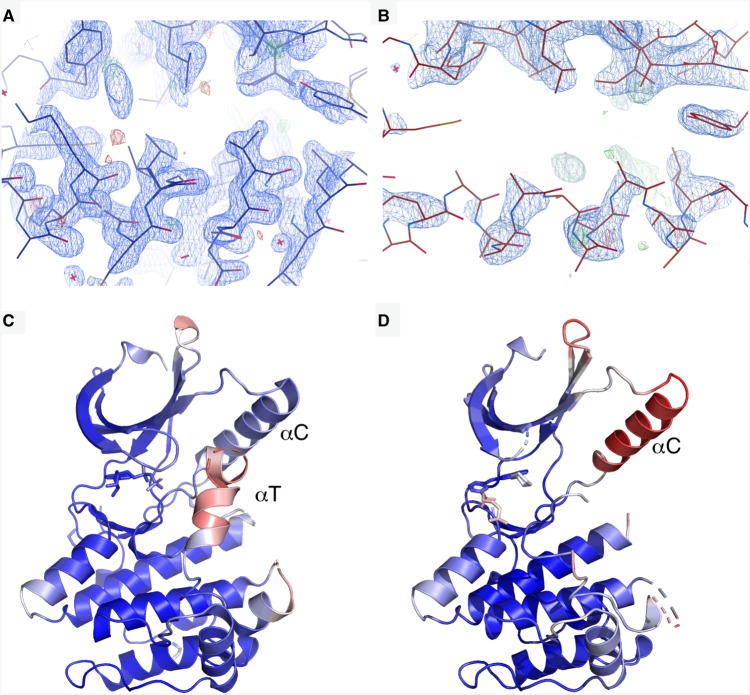
Exchange of ADP for compound 51 destabilises the C-helix of Nek2. View of the C-helix of (**a**) Nek2.ADP and (**b**) Nek2.**51** with 2Fo-Fc and Fo-Fc maps shown contoured at 1.0 σ and ±3 σ, respectively. (**c**) Nek2.ADP coloured by B-factor from 6.3 Å^2^ (blue) to 84.3 Å^2^ (red). (**d**) Nek2.**51** coloured by B-factor from 20.4 Å^2^ (blue) to 121 Å^2^ (red).

A striking feature of the Nek2.**51** structure is the contrast between the highly ordered core of the kinase and the poorly ordered C-helix (Figure 7). While the structure of Nek2-ADP (PDB code 2W5A) exhibits a well-ordered C-helix, as evidenced by clear electron density (Figure 7a), the C-helix in the Nek2.**51** structure has very poor electron density (Figure 7b). The C-helix in the Nek2-ADP structure has B-factors similar to the rest of the kinase core (Figure 7c), whereas the C-helix in the Nek2.**51** structure has the highest B-factors of any region modelled in the structure (Figure 7d). There is no structural evidence that compound **51** destabilises the C-helix directly. Instead, it appears that it is unable to compensate for the loss of the stabilising effect of ADP on the C-helix. ADP stabilises the activation loop into a short helix (αT), which interacts with and stabilises the C-helix (Figure 7c) [37]. We previously observed that soaking of ATP analogues or ATP-competitive inhibitors into Nek2 crystals stabilise alternative conformations of the activation loop, which also interact with and stabilise the C-helix [23]. However, the purine compound forms no interactions with the activation loop, which remains disordered and does not stabilise the C-helix.

## Discussion

The description of hydrophobic spines provides a framework for understanding the mechanisms of kinase regulation [13,38]. Aurora-A, for example, is regulated through interactions with binding partners such as TPX2 and TACC3 that promote the formation of a stacked R-spine [39–41]. Aurora-A is unusual in having a polar residue (Gln185) at the RS3 position that organises a network of water molecules that are critical for activity [42]. Indeed, 52.5% of kinases have a Leu at the RS3 position, including most human Nek kinases and BRAF (Leu505), based on an updated alignment of kinase domains [43]. Mutation of BRAF Leu505 to either Phe or Met generates a constitutively active kinase that can act independent of activation loop phosphorylation or dimerisation [36]. Building on these observations, we generated a mutant of Nek7 with an all-aromatic R-spine that formed a partially stacked configuration in the co-crystal structure with compound **51**, in contrast with the disrupted and variable R-spine configurations observed in the structure of Nek7^WT^ crystallised in the same crystal form. However, the SRS mutations did not fully rescue the stacked configuration of the R-spine that is observed in structures of active kinases — the side chain of Phe180 was incorrectly positioned — and did not confer enhanced activity on Nek7^SRS^. This might be due to a requirement for activation loop phosphorylation (on Ser195) for full Nek7 activity. Alternatively, it might be possible to restore a fully stacked R-spine in unphosphorylated Nek7 through additional mutations, perhaps to generate a larger pocket in which to accommodate the side chain of Phe180 in a canonical DFG-in position, as this pocket has evolved to fit a leucine side chain in the native DLG sequence. It would also be interesting to explore whether a similar strategy could be employed to stabilise the R-spines of other protein kinases.

Mutation of the autoinhibitory residue Tyr97 is sufficient to generate a constitutively active Nek7 in cells, but is insufficient to stabilise an active conformation of Nek7 that crystallises with a stacked configuration of the R-spine [11,12]. This is because the Y97F mutation releases autoinhibition, allowing Nek7 autophosphorylation to occur. The physiological activation of Nek7 is via Nek9, which induces the release of autoinhibition through the back-to-back dimerisation of Nek7. Nek9 can also activate Nek7 through direct phosphorylation of the activation loop on S195. Nek7 phosphorylated on S195 through the incorporation of a genetically encoded phospho-Ser amino acid during translation shows that this is sufficient for activation, as this protein is not further activated by Nek9 [34]. We, therefore, presume that this state of Nek7 has a stacked R-spine, however, our attempts to crystallise pS195-Nek7 have been unsuccessful. Indeed, there are no published structures of a Nek kinase that is phosphorylated on the activation loop. However, we can predict how phosphorylation might activate the kinase based on the structures of other kinases such as CDK2 [44]. The activation loop of CDK2 is phosphorylated on Thr160, and the phosphate group is co-ordinated by three Arg side chains: Arg50 in the C-helix, Arg126 in the HRD motif and Arg150 in the activation loop (at position DFG+3). This network of interactions couples structural elements that comprise three out of four of the R-spine residues, and thus stabilises a stacked configuration. Nek7 has basic residues at the three equivalent positions (Lys81, Lys140 and Lys164, respectively), and we predict that these three residues co-ordinate the phosphate group on pSer195. In contrast, Plk1, which has an R-spine composed of all aromatic residues, has only one of these three residues (Arg175 in HRD), perhaps reflecting a reduced requirement for phosphorylation in a kinase that already has a strong R-spine.

Nek7 functions in several cellular signalling pathways: the regulation of mitotic spindle assembly, inflammasome activation, G1 progression, centriole duplication and protection of telomeres from oxidative damage [2,6–8,45–47]. Unfortunately, there are currently no compounds with the selectivity and potency required to be a useful chemical tool to probe Nek7 function in cells. Screening of panels of kinase inhibitors against Nek7 and the closely related Nek6 has yielded hit rates of less than 5%, low compared with most kinases [22]. In their broad survey of kinase inhibitors, Drewry et al. [48] identified Nek7 as a ‘gap’ kinase for which no potent and selective inhibitor were available. However, this study also identified 11 compounds that inhibited Nek7 more than 80% at 1µM, including three compounds that inhibited Nek7 at 100%, using a single KINOMEscan assay experiment that screened 645 compounds against 468 kinases. The top three compounds belong to three distinct chemical classes and had variable levels of selectivity: the 5-Ar-indazole inhibited 71 kinases at >90%, the 4-anilinoquinoline inhibited only eight kinases at >90% and the imidazotriazine inhibited 12 kinases at > 90%. Other compounds of interest from recent studies include a series of JNK inhibitors based on an aminopyrimidine scaffold that inhibit Nek7 at >80% at 10 µM compound and a GSK-3 inhibitor based on an aminopyrazole scaffold that inhibits Nek7 > 60% at 0.625 µM [22,49]. Although IC_50_ data are not available for these compounds, the single-point activities suggest that they are more active against Nek7 than compound **51**. There is, therefore, a growing set of starting points to obtain Nek7 inhibitors and the structure of compound **51** bound to Nek7 provides a template for the structure-based design of more potent and selective inhibitors of this kinase.

## Abbreviations

Neks: NIMA-related kinases
R-spine: regulatory-spine
